# Factors affecting mortality among HIV positive patients two years after completing recommended therapy for Cryptococcal meningitis in Uganda

**DOI:** 10.1371/journal.pone.0210287

**Published:** 2019-01-30

**Authors:** Jonathan Kitonsa, Yunia Mayanja, Emmanuel Aling, Julius Kiwanuka, Juliana Namutundu, Zacchaeus Anywaine, Abu-Baker Ggayi, Freddie Kibengo, Noah Kiwanuka, Pontiano Kaleebu

**Affiliations:** 1 Medical Research Council / Uganda Virus Research Institute & London School of Hygiene and Tropical Medicine Uganda Research Unit, Entebbe, Uganda; 2 AIDS Healthcare Foundation, Kampala, Uganda; 3 Department of Epidemiology and Biostatistics, School of Public Health, Makerere University College of Health Sciences, Kampala, Uganda; Public Health England, UNITED KINGDOM

## Abstract

**Background:**

Cryptococcal meningitis (CCM) remains a leading cause of mortality amongst HIV infected patients in sub-Saharan Africa. When patients receive recommended therapy, mortality at 10 weeks has been reported to vary between 20 to 36%. However, mortality rate and factors affecting mortality after completing recommended therapy are not well known. We investigated mortality rate, and factors affecting mortality at 2 years among CCM patients following completion of recommended CCM therapy in Uganda.

**Methods:**

A retrospective cohort study was conducted among HIV infected patients that had completed 10 weeks of recommended therapy for CCM (2 weeks of intravenous amphotericin B 1mg/kg and 10 weeks of oral Fluconazole 800mg daily) in the CryptoDex trial (ISRCTN59144167) between 2013 and 2015. Survival analysis applying Cox regression was used to determine the mortality rate and factors affecting mortality at 2 years.

**Results:**

This study followed up 112 participants for 2 years. Mean age (±SD) was 34.9 ± 8, 48 (57.1%) were female and 80 (74.8%) had been on ART for less than 1 year. At 2 years, overall mortality was 30.9% (20 deaths per 100 person-years). Majority of deaths (61.8%) occurred during the first 6 months. In multivariable analysis, mortality was associated with ever being re-admitted since discharge after hospital-based management of CCM (aHR = 13.33, 95% CI: 5.92–30.03), p<0.001; and self-perceived quality of life, with quality of life 50–75% having reduced risk compared to <50% (aHR = 0.21, 95% CI: 0.09–0.5), p<0.001, as well as >75% compared to <50% (HR = 0.29, 95% CI: 0.11–0.81), p = 0.018.

**Conclusion:**

There remains a considerable risk of mortality in the first two years after completion of standard therapy for CCM in resource-limited settings with risk highest during the first 6 months. Maintenance of patient follow up during this period may reduce mortality.

## Introduction

Mortality from HIV related opportunistic infections remains high especially in sub-Saharan Africa despite widespread antiretroviral therapy (ART) use [1]. Cryptococcal meningitis (CCM) is one of the leading contributors, killing as many as 70% of infected people especially in settings where treatment is inadequate [1–3]. In 2014, 162,500 cases of CCM were estimated to occur in sub-Saharan Africa annually, leading to 135,900 deaths [1]. Uganda ranked fourth on incidence in the region with 12,200 cases estimated to occur annually, only lower than Nigeria (27,100 cases), South Africa (21,400 cases) and Mozambique (18,600 cases). One of the reasons for the high mortality in CCM in low resource countries including Uganda is that majority of the patients are treated with suboptimal regimens, in most cases using varying doses of Fluconazole [4]. However, even when treated with optimal regimens (Amphotericin based therapy), mortality in these settings has remained high, varying between 20% and 36% at 10 weeks due to a number of reasons such as elevated intracranial pressure, inadequate fungal clearance and drug-related toxicities [5–7].

Little is known about the mortality rate and factors affecting mortality after completion of standard treatment for CCM. These patients remain debilitated by complications such as visual and hearing impairment, movement abnormalities and convulsions, among others. They also remain at risk of CCM relapse and immune reconstitution syndrome (IRIS) [7,8]. These may cause the risk of mortality to remain. Here, we present results of a study conducted to determine the mortality rate and factors affecting mortality among patients that had completed 10 weeks of treatment for CCM in Uganda.

## Methodology

### Study design and setting

This was a retrospective cohort study conducted among patients that had completed 10 weeks of treatment for CCM in Uganda in a previous trial—the CryptoDex trial (Clinical trials registration number ISRCTN59144167) [9]. The CryptoDex trial was a double-blind placebo-controlled phase III trial of adjunctive dexamethasone in HIV infected adults with Cryptococcal meningitis. The trial was conducted at six sites in Asia and Africa including two sites in Uganda between 2013 and 2015. The Ugandan sites were located at the Medical Research Council/Uganda Virus Research Institute and London School of Hygiene and Tropical Medicine Uganda Research Unit (MRC/UVRI & LSHTM Uganda Research Unit) clinics, in Masaka and Entebbe towns. Entebbe and Masaka are located 37 and 140 kilometres respectively south-west of Kampala city. Patients enrolled from Masaka were admitted to Masaka Regional Referral Hospital and mainly originated from rural settings that make up the hospital catchment area. Patients enrolled at Entebbe were admitted to Entebbe General Hospital and originated from semi-urban settings that serve as the catchment area for the hospital. Both hospitals are public facilities. This study only collected information on patients previously managed in Uganda partly because of resource constraints but also because this country represents the highest burden of CCM among those involved in the CryptoDex study.

### Patients enrolled in the CryptoDex trial and their management

Patients recruited in the CryptoDex trial were 18 years and above, had a clinical syndrome consistent with HIV associated CCM, and microbiologically confirmed disease as indicated by one or more of the following test results: positive India ink staining of cerebral spinal fluid (CSF); culture of Cryptococcal species from CSF or blood; or Cryptococcal antigen detected in CSF on Cryptococcal antigen lateral flow assay. They were randomised to receive either dexamethasone adjunctive therapy or placebo in a tapered dose up to 42 days post randomisation. All patients were treated with intravenous Amphotericin B (1mg/kg/day) and 800mg daily oral Fluconazole for 14 days (induction phase), followed by 800mg daily Fluconazole (continuation phase) to complete 10 weeks of treatment. After 10 weeks, all patients were switched to 200mg daily Fluconazole (Maintenance phase). This regimen is recommended by WHO for settings where Flucytosine is not available to be used during the induction period [10].

### Study subjects and sampling for the two-year post CCM treatment study

The two-year post CCM treatment study was conducted between May 2017 and July 2017 (data collection), and collected information on participants that had survived up to 10 weeks in the CryptoDex trial, and therefore completed 10 weeks of treatment. Participants whose survival status could not be determined for at least one time point after completion of treatment in the CryptoDex trial were ineligible. Following this eligibility criterion, all 112 participants that completed 10 weeks in the CryptoDex trial qualified to participate in this study and were recruited.

### Data collection

Data was collected using semi-structured questionnaires, which were developed in English, translated to Luganda and back-translated. These were standardised and initially piloted prior to use in this study to assess comprehensibility. Four trained research assistants administered the questionnaires. Information from participants who died after 10 weeks in the study was acquired through a next of kin while living participants were contacted in person. Participants were traced using contact details previously collected while in the CryptoDex trial. One session was organised with the participant or next of kin during which an interview was administered while other required information was retrieved from the CryptoDex trial database.

### Variables and their measurement

The dependent variable was time to death measured from the date a participant completed 10 weeks in the CryptoDex trial. Independent variables included demographic characteristics (age, sex, marital status, education); site i.e. Masaka (rural) or Entebbe (semi-urban); quality of life at completion of week 10 in the CryptoDex trial (measured using the EQ VAS scale of the EQ-5D-3L tool [11], presented as a percentage of self-perceived quality of life relative to full health); antiretroviral therapy (ART) use at treatment completion (on ART or not on ART); readmission status (ever readmitted or never readmitted, i.e., a patient was discharged when stable after completing intravenous therapy in the CryptoDex trial, this variable referring to re-admission that occurred between discharge from hospital and completion of 10 weeks of therapy); and randomisation status as received dexamethasone or not. Other information collected included the date of completion of 10 weeks in the CryptoDex trial, location where participant died and most likely cause of death based on a verbal autopsy (conducted using a tool adapted from the COSTOP trial [12]) complemented by information from patient records where accessible.

### Data analysis

Data was entered into a Microsoft Access database, cleaned and exported to STATA (College Station, TX, version 13.0). Descriptive statistics were presented in form of frequencies and percentages for categorical variables while continuous variables were summarised using means (standard deviation) and/or medians (interquartile ranges).

Data was then converted into survival format, i.e., time-to-event data. The date the participant completed 10 weeks of treatment for CCM served as time zero/entry time while censoring was done at the time a participant completed 2 years from entry or the time last seen for those we could not trace. Time accrued was measured in person-years estimated as the difference in time from entry to death or time of censor. A Kaplan-Meier (K-M) graph was computed for the cohort to demonstrate the survival trend over the two years. The life table method was used to compute the proportion surviving at different intervals of time. Overall mortality rate was determined, followed by mortality stratified by participant characteristics. Bivariate analysis was done to determine crude associations between mortality and participant characteristics by fitting Cox regression models. Variables that had a p-value of 0.2 and below at bivariate analysis were used in building a multivariable Cox regression model using a stepwise approach. Age and Sex were tested in the model irrespective of their p-value at bivariable analysis. The proportional hazards assumption was tested for all variables used in the final model. The same variables were also tested for inter-associations using logistic regression. In the final model, all variables with a p-value of 0.05 and below were considered statistically significant. Results are reported as hazard ratios (HR) and their 95% confidence intervals (CI).

### Ethical considerations

The study was approved by the Makerere University School of Public Health Research and Ethics Committee. Permission to use information from the CryptoDex database was acquired following standard MRC/UVRI & LSHTM Uganda Research Unit data sharing policy available at https://www.mrcuganda.org/publications/data-sharing-policy. Written informed consent was obtained from all participants prior to data collection. For participants that had died, a next of kin provided consent.

## Results

### Baseline characteristics

**Fig 1:**
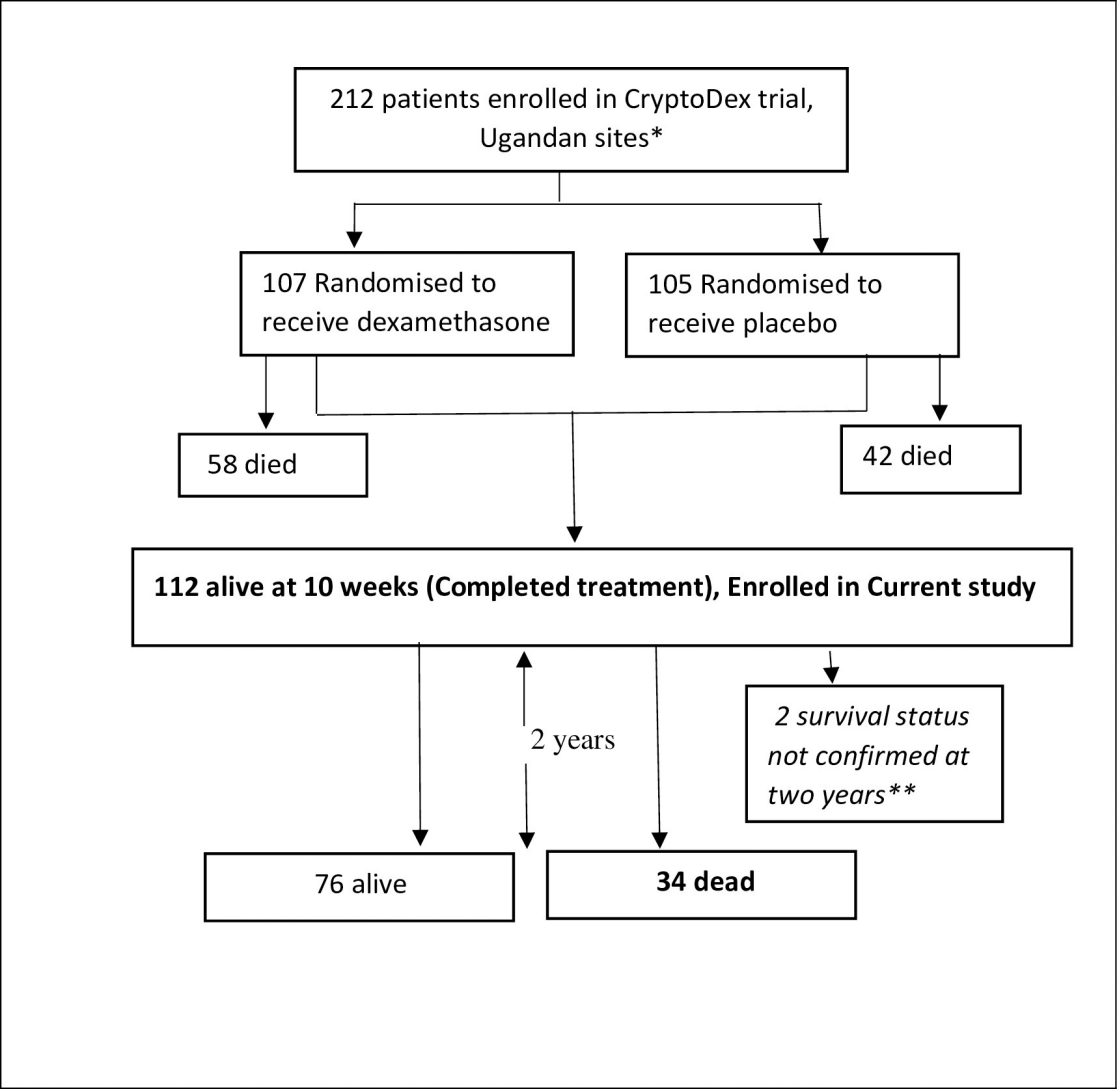
Flow diagram showing participants enrolled into current study from the CryptoDex trial. *All patients treated with 1ml/kg of intravenous amphotericin B for 2 weeks and 800mg of oral Fluconazole daily for 10 weeks. **Participants last seen before completion of 2 years.

Of the 212 participants enrolled in the CryptoDex trial in Uganda, 100 died while 112 completed 10 weeks of therapy. All 112 participants that had completed treatment were found eligible for this study and were included (Fig 1). Forty-two (37.5%) of these had been managed at the Entebbe site and 70 (62.5%) at the Masaka site. The mean age (±SD) of participants was 34.9 ± 8 years and 56 (50%) were between 30–40 years. Sixty-four (57.1%) of the participants were female and 104 (92.9%) had attained at least primary level education. The median duration since HIV diagnosis was 0.8 years (IQR 0.3, 2.8), with 61 (54.5%) diagnosed within one year prior to completion of CCM treatment. The median duration on ART at baseline was 3.3 months (IQR 1.2, 11.1) with 80 of 107 (74.8%) having been on ART for less than 1 year. Thirty-five participants (31.2%) rated their baseline quality of life to be above 75%, while 60 (53.6%) were between 50–75%. Eighty-one participants (73.6%) had not been re-admitted between the admission at CCM diagnosis and completion of therapy at 10 weeks. Details of the baseline characteristics are shown in Table 1.

**Table 1:** Baseline characteristics of study participants at completion of Cryptococcal meningitis therapy.

Characteristics	Category	Frequency	Percentage
Site	Entebbe (Semi-urban)	42	37.5
	Masaka (Rural)	70	62.5
Age in years	<30	32	28.6
	30–40	56	50.0
	41+	24	21.4
Sex	Male	64	57.1
	Female	48	42.9
Marital Status	Married	51	45.5
	Not Married	61	54.5
Education	None	8	7.1
	Primary	69	61.6
	Post primary	35	31.3
Duration since HIV diagnosis (years)	<1	61	54.5
	1–5	25	22.3
	>5	26	23.2
ART status at Baseline	On ART	107	95.5
	Not on ART	5	4.5
Duration on ART (months)	<3	46	43.0
	3–12	34	31.8
	>12	27	25.2
Baseline quality of life (%)	<50	17	15.2
	50–75	60	53.6
	75+	35	31.2
Dexamethasone use	Yes	49	43.8
	No	63	56.2
Readmission status	Ever readmitted	31	27.7
	Never readmitted	81	72.3

**Abbreviations**: SD, standard deviation; IQR, interquartile range; ART, Antiretroviral therapy.

### Mortality rate at 2 years

**Fig 2:**
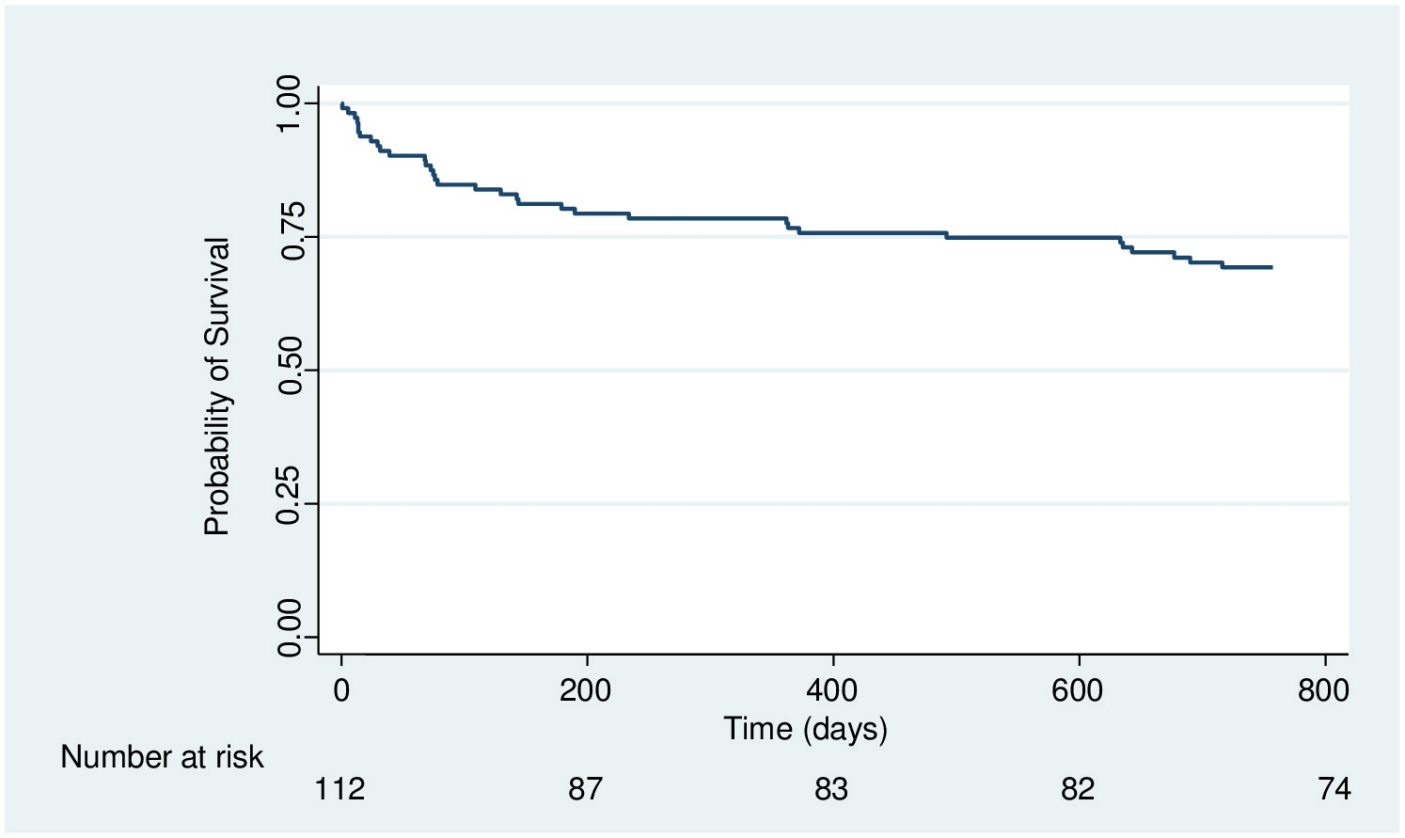
Kaplan-Meier plot showing survival of participants during two years of follow up after completion of recommended therapy.

At 2 years following completion of treatment for CCM, 34 of the 112 participants (30.4%) had died, giving an overall mortality rate of 20 deaths per 100 person-years after 172.7 person-years of follow up. Fig 2 is a K-M plot showing the survival of the whole cohort in the two-year period. The proportion surviving had reduced to 81.2% by 6 months and from there only reduced to 70.2 at 24 months basing on the life-table (61.8% of all deaths occurred in the first 6 months). Fifty percent (17) of the deaths occurred at home and the rest at a health-care facility.

Of 28 participants for whom a probable cause of death could be established, eight had features suggestive of either CCM relapse or IRIS, six died due to septicaemia, four due to dehydration and two due to Anaemia. Other causes of death included hypoglycaemia, renal failure, cancer, Addisonian crisis and HIV wasting syndrome.

### Factors affecting mortality

Table 2 shows the unadjusted and adjusted hazard ratios for the different factors evaluated. All variables used in the final model met the proportional hazards assumption (p = 0.76 for site, p = 0.9 and p = 0.68 for quality of life <50 compared with 50–75 and <50 compared to 75+ respectively, and p = 0.13 for readmission status). At multivariable analysis, ever being re-admitted after CCM management was associated with increased risk of mortality (aHR = 13.33, 95% CI: 5.92–30.03), p<0.001. There was reduced risk of mortality comparing quality of life 50–75% and <50% (aHR = 0.21, 95% CI: 0.09–0.5), p<0.001, as well as quality of life >75% compared to <50% (HR = 0.29, 95% CI: 0.11–0.81), p = 0.018. There was no significant relationship between any two of the three variables used in the final model, i.e., site, baseline quality of life, and readmission status.

**Table 2:** Mortality rates, bivariate and multivariate analysis showing factors affecting mortality.

Variable	Categories	Number of deaths/ Person-time at risk	Mortality rates (per 100pyrs)	Unadjusted Hazard Ratios (95% CI)	P-value	Adjusted Hazard Ratios (95% CI)	P-value**
Overall		34/172.7	20				
Site	Entebbe	16/61.8	26	1		1	
	Masaka	18//110.9	16	0.64 (0.33–1.25)	0.19	0.48(0.22–1.03)	0.06
Age (yrs.)	<30	12/49.5	24	1			
	30–40	17/84.7	20	0.82 (0.39–1.72)	0.60		
	41+	5/38.5	13	0.56 (0.20–1.58)	0.27		
Sex	Male	19/97.9	19	1			
	Female	15/74.8	20	1.03 (0.53–2.03)	0.92		
Marital Status	Married	18/72.7	25	1			
	Not Married	16/99.4	16	0.67 (0.34–1.32)	0.25		
Duration since HIV^+^ diagnosis (yrs.)	<1	20/88.9	23	1			
	1–5	7/41.2	17	0.79 (0.33–1.87)	0.60		
	>5	7/42.6	16	0.75 (0.32–1.77)	0.51		
ART status	Pre-ART	1/4.4	23	1			
	On ART	33/168.2	20	0.67 (0.31–1.43)	0.29		
Duration on ART (months)	<3	14/75.1	19	1			
	3–12	14/49.0	29	1.47 (0.70–3.09)	0.31		
	>12	5/44.2	11	0.62 (0.22–1.72)	0.36		
Baseline quality of life	<50%	10/19.2	52	1		1	
	50–75%	14/93.3	15	0.28 (0.13–0.64)	0.002	0.21 (0.09–0.5)	<0.001*
	76+%	10/55.5	18	0.35 (0.14–0.83)	0.019	0.29 (0.11–0.81)	0.018*
Received dexamethasone	No	21/91.3	23	1			
	Yes	13/81.3	16	0.69 (0.34–1.40)	0.30		
Re-admission status	Never readmitted	12/145.7	8	1		1	
	Ever readmitted	22/26.9	82	7.84 (3.84–16.05)	<0.001	13.33 (5.92–30.04)	<0.001*

Abbreviations: Pyrs, Person-years; CI, Confidence interval; ART, Antiretroviral therapy; HIV, Human immunodeficiency virus; yrs., years

* P-value clinically significant at the 5% level (in the adjusted model).

** P-values represented are for variables that had a p-value <0.2 in bivariable analysis that were used in the final adjusted model.

## Discussion

Mortality in this cohort of patients that had completed recommended treatment for CCM was high, most remarkably during the first six months. Most studies previously done on CCM have evaluated mortality from the time of treatment initiation or diagnosis [5,13,14]. This cohort of AIDS patients manifest higher mortality compared to AIDS patients with similar immunological status in Uganda. In a study done amongst Ugandan AIDS patients without cryptococcosis, the mortality rate was much lower at 3.68 and 2.75 per 100 person-years among groups with CD4 between 50–99 and 100–149 cells/uL respectively [14]. High mortality was demonstrated in another study done by Butler. E et al, where out of 104 patients alive at 10 weeks of CCM treatment, 30 had died approximately 22 months later, a proportionate mortality of 29% [13]. The same study revealed that mortality considered from the time of treatment initiation is highest during the first 6 months [13]. Since our study determined mortality starting from the time of treatment completion (10 weeks), the six months spurn considered by Butler. E et al include close to 4 months of our first 6 months. These findings affirm that the threat of mortality does not necessarily disappear at treatment completion, but extends for some time. With majority of previous studies emphasising the need to identify means of reducing mortality during the 10 weeks of treatment [15–19], this excludes the considerable number of deaths that occur in the first 6 months after treatment completion.

Verbal autopsy suggested that many of the deaths were likely to be attributable to CCM relapse or IRIS. IRIS has been reported to increase the risk of mortality among CCM patients initiating ART. In a study done by Boulware D et al among CCM patients in Uganda, those who developed IRIS were 2.5 times more likely to die than the rest of the cohort, with IRIS occurring at a median of 8.8 weeks from ART initiation [20]. Majority of participants in our study had been on ART for less than 3 months, a factor that could have increased their risk of getting IRIS. Relapse could have resulted from non-continuation of or poor adherence to Fluconazole maintenance therapy since this is often unavailable in health facilities offering care to HIV infected patients in Uganda given its high cost.

According to Tenforde M et al, the occurrence of Cryptococcal meningitis is an indication of an unsuccessful HIV care cascade. They further suggest that lack of follow up of patients after treatment for CCM may partly be responsible for the bad treatment outcomes observed in resource-limited settings [7]. The presence of a hospital or health facility offering care and treatment for CCM and completion of treatment thereof, may not guarantee survival thereafter. Some patients still go back to the same conditions where they came from prior to diagnosis, which may be characterised by inadequacies in the social-economic conditions and poor health services. These may therefore remain at risk of unfavourable outcomes such as IRIS and relapse [7,8] for several months after completion of standard therapy. Even those that go to ART providing centres may not get the kind of care they would have required for their post-CCM sequelae due to lack of adequate logistics and health worker skills, and this may eventually contribute to post-treatment mortality [7].

We found that low self-perceived quality of life was associated with higher mortality. The EQ-VAS scale used in this study gave respondents the opportunity to determine their own health status at that particular point relative to what one considered as their own ideal (100%). According to Jeremy D et al, successful treatment of CCM depends not only on effective antifungal therapy but also on successful management of complications [9]. Other studies have also shown that CCM survivors remain with many disabilities [4,15]. These complications and disabilities do not necessarily cease at treatment completion, but continue and compromise a patient’s quality of life, thereby posing a risk to the patients’ life [21]. An effect of quality of life on survival has been demonstrated elsewhere in a study done in the United States among HIV infected patients [22]. Ever being re-admitted after hospitalisation for CCM management was also a risk factor for mortality. This suggests that those ever re-admitted are another vulnerable group that needs extra follow up to curb the risk of mortality. It is interesting to note that dexamethasone adjunctive therapy did not significantly affect mortality at 2 years among those that had completed CCM therapy. In the CryptoDex trial, dexamethasone did not have an effect on the risk of mortality at 10 weeks [23]. In addition to the reasons proposed in the CryptoDex paper for this observation, we did not expect patients that had received dexamethasone to have residual drug in their system, because approximately one month had elapsed between the last dose of dexamethasone and completion of 10 weeks of therapy for CCM. This drug has a short half-life of 1–5 hours.

Because this study used pre-collected data, information on some important variables such as ART adherence and Fluconazole continuation status could not be established. It is known that Fluconazole continuation reduces the risk of relapse which may have an effect on mortality [24]. However, it is important to appreciate that this information is not available yet at treatment completion and therefore cannot inform the clinician’s decision to determine the nature of follow up required. This study considered all-cause mortality. Exclusion of mortality attributable to other causes could have given a better appreciation of the associations demonstrated here, or revealed more associations we were not able to find. However, this being an immunologically depleted group of patients, they are at risk of other opportunistic infections and therefore follow up ought to look out for these as well. Lastly, we had a small sample size, potentially limiting the number of variables that could be evaluated. However, we had a reasonable number of outcomes to do a modest multivariable analysis. This study did well to follow up a cohort of patients that have previously not received a lot of attention. These are patients that everyone would have expected to be well since they had completed recommended treatment. To the contrary, we reveal that this remains a special group that may need more than routine care. These findings are likely to be generalizable to HIV positive patients completing treatment for CCM in resource-limited settings.

This study reveals that patients that have completed treatment for CCM remain at risk of mortality and therefore need follow up especially during the first six months. Patients that have low quality of life (<50) and those that have ever been readmitted need to be more closely followed up. HIV care facilities in sub-Saharan Africa need to be restructured so that they are in position to offer specialised care for patients previously treated for CCM, a disease that carries a huge burden of mortality during and after treatment.

## Supporting information

S1 Dataset (XLSX)

S1 Questionnaire (PDF)

S2 Questionnaire (PDF)

S1 Data extraction checklist (PDF)

## References

[1] Rajasingham R, Smith RM, Park BJ, Jarvis JN, Govender NP, Chiller TM, et al (2017) Global burden of disease of HIV-associated cryptococcal meningitis: an updated analysis. The Lancet Infectious Diseases 17: 873–881. 10.1016/S1473-3099(17)30243-8 28483415 PMC5818156

[2] Gordon SB, Walsh AL, Chaponda M, Gordon MA, Soko D, Molyneux ME, et al (2000) Bacterial Meningitis in Malawian Adults: Pneumococcal Disease is Common, Severe, and Seasonal. Clinical Infectious Diseases 31: 53–57. 10.1086/313910 10913396

[3] Park BJ, Wannemuehler KA, Marston BJ, Govender N, Pappas PG, Chiller TM. (2009) Estimation of the current global burden of cryptococcal meningitis among persons living with HIV/AIDS. AIDS 23: 525–530. 10.1097/QAD.0b013e328322ffac 19182676

[4] Day JN, Chau TTH, Wolbers M, Mai PP, Dung NT, Mai H, et al (2013) Combination Antifungal Therapy for Cryptococcal Meningitis. New England Journal of Medicine 368: 1291–1302. 10.1056/NEJMoa1110404 23550668 PMC3978204

[5] Kambugu A, Meya DB, Rhein J, O’Brien M, Janoff EN, Ronald AR, et al (2008) Outcomes of Cryptococcal Meningitis in Uganda Before and After the Availability of Highly Active Antiretroviral Therapy. Clinical Infectious Diseases 46: 1694–1701. 10.1086/587667 18433339 PMC2593910

[6] Sloan DJ, Parris V (2014) Cryptococcal meningitis: epidemiology and therapeutic options. Clin Epidemiol 6: 169–182. 10.2147/CLEP.S38850 24872723 PMC4026566

[7] Tenforde MW, Wake R, Leeme T, Jarvis JN (2016) HIV-Associated Cryptococcal Meningitis: Bridging the Gap Between Developed and Resource-Limited Settings. Current Clinical Microbiology Reports 3: 92–102. 10.1007/s40588-016-0035-5 27257597 PMC4845086

[8] Bicanic T, Meintjes G, Rebe K, Williams A, Loyse A, Wood R, et al (2009) Immune Reconstitution Inflammatory Syndrome in HIV-Associated Cryptococcal Meningitis: A Prospective Study. JAIDS Journal of Acquired Immune Deficiency Syndromes 51: 130–134. 10.1097/QAI.0b013e3181a56f2e 19365271

[9] Day J, Imran D, Ganiem AR, Tjahjani N, Wahyuningsih R, Adawiyah R, et al (2014) CryptoDex: A randomised, double-blind, placebo-controlled phase III trial of adjunctive dexamethasone in HIV-infected adults with cryptococcal meningitis: study protocol for a randomised control trial. Trials 15: 441 10.1186/1745-6215-15-441 25391338 PMC4289250

[10] Sloan DJ, Parris V (2014) Cryptococcal meningitis: epidemiology and therapeutic options. Clinical Epidemiology 6: 169–182. 10.2147/CLEP.S38850 24872723 PMC4026566

[11] The EuroQol Group (1990) EuroQol-a new facility for the measurement of health-related quality of life. Health policy 16: 199–208. 10109801 10.1016/0168-8510(90)90421-9

[12] Anywaine Z, Abaasa A, Levin J, Kasirye R, Kamali A, Grosskurth H, et al (2015) Safety of discontinuing cotrimoxazole prophylaxis among HIV infected adults on anti-retroviral therapy in Uganda (COSTOP trial): Design. Contemporary Clinical Trials 43: 100–104. 10.1016/j.cct.2015.05.015 26009024 PMC4542218

[13] Butler EK, Boulware DR, Bohjanen PR, Meya DB (2012) Long Term 5-Year Survival of Persons with Cryptococcal Meningitis or Asymptomatic Subclinical Antigenemia in Uganda. PLOS ONE 7: e51291 10.1371/journal.pone.0051291 23251485 PMC3519582

[14] Mills EJ, Bakanda C, Birungi J, Chan K, Ford N, Cooper CL, et al (2011) Life expectancy of persons receiving combination antiretroviral therapy in low-income countries: A cohort analysis from uganda. Annals of Internal Medicine 155: 209–216. 10.7326/0003-4819-155-4-201108160-00358 21768555

[15] van der Horst CM, Saag MS, Cloud GA, Hamill RJ, Graybill JR, Dedicoat M. (1997) Treatment of Cryptococcal Meningitis Associated with the Acquired Immunodeficiency Syndrome. New England Journal of Medicine 337: 15–21. 10.1056/NEJM199707033370103 9203426

[16] Muzoora CK, Kabanda T, Ortu G, Ssentamu J, Hearn P, Mwesigye J, et al (2012) Short course amphotericin B with high dose fluconazole for HIV-associated cryptococcal meningitis. Journal of Infection 64: 76–81. 10.1016/j.jinf.2011.10.014 22079502

[17] Sloan D, Dlamini S, Paul N, Dedicoat M (2008) Treatment of acute cryptococcal meningitis in HIV infected adults, with an emphasis on resource-limited settings. Cochrane Database of Systematic Reviews. 10.1002/14651858.CD005647.pub2 18843697

[18] Jackson A (2012) A Phase II Randomised Controlled Trial Adding Oral Flucytosine to High Dose Fluconazole, with Short-course Amphotericin B, for Cryptococcal Meningitis. 26: 1363–1370. 10.1097/QAD.0b013e328354b419 PMC3776948 22526517

[19] Loyse A, Wilson D, Meintjes G, Jarvis JN, Bicanic T, Bishop L, et al (2012) Comparison of the Early Fungicidal Activity of High-Dose Fluconazole, Voriconazole, and Flucytosine as Second-Line Drugs Given in Combination With Amphotericin B for the Treatment of HIV-Associated Cryptococcal Meningitis. Clinical Infectious Diseases 54: 121–128. 10.1093/cid/cir745 22052885

[20] Boulware DR, Meya DB, Bergemann TL, Wiesner DL, Rhein J, Musubire A, et al (2010) Clinical Features and Serum Biomarkers in HIV Immune Reconstitution Inflammatory Syndrome after Cryptococcal Meningitis: A Prospective Cohort Study. PLOS Medicine 7: e1000384 10.1371/journal.pmed.1000384 21253011 PMC3014618

[21] Davis S (2004) Clinical Sequelae Affecting Quality of Life in the HIV-Infected Patient. Journal of the Association of Nurses in AIDS Care 15: 28S–33S. 15587606 10.1177/1055329004269478

[22] Cunningham WE, Crystal S, Bozzette S, Hays RD (2005) The Association of Health-related Quality of Life with Survival Among Persons with HIV Infection in the United States. Journal of General Internal Medicine 20: 21–27. 10.1111/j.1525-1497.2005.30402.x 15693923 PMC1490035

[23] Beardsley J, Wolbers M, Kibengo FM, Ggayi A-BM, Kamali A, Cuk NTK, et al (2016) Adjunctive Dexamethasone in HIV-Associated Cryptococcal Meningitis. New England Journal of Medicine 374: 542–554. 10.1056/NEJMoa1509024 26863355 PMC4778268

[24] Bozzette SA, Larsen RA, Chiu J, Leal MAE, Jacobsen J, Rothman P, et al (1991) A Placebo-Controlled Trial of Maintenance Therapy with Fluconazole after Treatment of Cryptococcal Meningitis in the Acquired Immunodeficiency Syndrome. New England Journal of Medicine 324: 580–584. 10.1056/NEJM199102283240902 1992319

