# Supplementary material for: Factors affecting mortality among HIV positive patients two years after completing recommended therapy for Cryptococcal meningitis in Uganda
Source: PLoS One. 2019 Jan 30;14(1):e0210287. doi: 10.1371/journal.pone.0210287 (PMC6353088; doi:10.1371/journal.pone.0210287)
Supplement: S1 Questionnaire — (PDF) [file pone.0210287.s002.pdf]

EVALUATION OF SURVIVAL OF HIV POSITIVE PATIENTS FOLLOWING STANDARD TREATMENT FOR CRYPTOCOCCAL MENINGITIS (CCM) IN UGANDA.

Questionnaire (English)

1) Demographics

i) Patient ID ……………………………………………………………………………

ii) Gender
   1) Male
   2) Female

iii) D.O.B / Age (dd/mmm/yyyy):
   ………………………………………………………………………………………

iv) Maximum education attained (class) /number of years in school: ……………………

v) Marital status
   a) Married
   b) Separated
   c) Single.

2) Is the patient still alive?

a) Yes

b) No.

3) If Dead,

i) What was the date of death?

………………………………………………………………………..

ii) If records available,
what was the diagnosis at time of death?

…………………………………………………………………

Or, if records not available,
what were the 5 most remarkable symptoms prior to death?

………………………………..
………………………………..
………………………………..
…………………………………
………………………………...

iii) Where did the patient die from?

a) Home
b) Hospital/health facility (Write hospital / health centre name here ……………………………………)

iv) Was the patient taking fluconazole prior to death?

a) No
b) Yes, but inconsistently
c) Yes and consistently

v) Was patient taking ART?

a) No
b) Yes, but inconsistently
c) yes and consistently.

vi) How often was a caretaker available during a week?

a) 5-7 days a week
b) 2-4 days a week
c) Less than 2 days a week.

vii) How was the care taker related to the patient?

a) Wife/Husband
b) Mother/Father
c) Sibling
d) Other, specify……………………………………………………..

viii) Was the patient ever readmitted since the initial treatment for meningitis to the time of completion of 10 weeks' of treatment?

a) No
b) Yes

ix) If answer to viii) is (yes), how many times was the patient readmitted?

a) Once
b) 2 times
c) 3 times
d) More than 3 times.

4) If the patient is still alive,

i) Is the patient still taking fluconazole?

a) No

b) Yes, but inconsistently

c) Yes and consistently

ii) If answer to i) above is no, why is patient not taking fluconazole

a) Stopped by clinician
b) Drugs not available at HIV clinic
c) Patient choice

iii) Is patient taking ART?

a) No
b) Yes, but inconsistently
c) Yes and consistently.

iv) Does the patient have a caretaker?

a) Yes
b) No

If answer is (No), go to (vii)

v) How is the caretaker related to the patient?

a) Wife/Husband
b) Mother/Father
c) Sibling
d) Other, specify…………………………………………………………..

vi) How often is a caretaker available during a week?

vi) 5-7 days a week
vii) 2-4 days a week
viii) Less than 2 days a week.

vii) Has the patient ever been readmitted?

a) Yes
b) No

viii) If yes, how many times?

……………………………………

ix) Has the patient resumed work? (This could be the work he/she used to do prior to being admitted or any new activity that supports their livelihood).

a) Yes
b) No

x) If answer to (viii) above is "yes", what type of work does the patient do?

………………………………………………………………………………………
……………………....
