# Supplementary material for: Factors affecting mortality among HIV positive patients two years after completing recommended therapy for Cryptococcal meningitis in Uganda
Source: PLoS One. 2019 Jan 30;14(1):e0210287. doi: 10.1371/journal.pone.0210287 (PMC6353088; doi:10.1371/journal.pone.0210287)
Supplement: S2 Questionnaire — (PDF) [file pone.0210287.s003.pdf]

EVALUATION OF SURVIVAL OF HIV POSITIVE PATIENTS FOLLOWING STANDARD TREATMENT FOR CRYPTOCOCCAL MENINGITIS (CCM) IN UGANDA.

Questionnaire (Luganda)

1) Ebikwaata ku mwetabi

i) Ennamba y’okunonyereza
………………………………………………………………………………

ii) Obuzaale
1) Musajja
2) Mukazi

iii) Ennaku z’obuzaale / Emyaka (dd/mmm/yyyy):
…………………………………………………………………………………………

iv) Ekibiina kyeyakomamu mu kusoma/ Emyaka gyeyamala mu ssomero:
……………………

v) Embeera kuby’obufumbo
a) Mufumbo
b) Yayawukana okuva mu bufumbo
c) Tafumbirwangako.

2) Omulwadde akyali mulamu?

a) Ye

b) Nedda.

3) Bwaba yafa,

i) Yafa nnaku za mwezi ki?
………………………………………………………………………..

ii) Ebiwandiiko bwebibaawo,
Yafa bulwadde ki? ………………………………………………………….
Oba, ebiwandiiko bwebitabaawo,
bubonero ki obwasinga okweyoleka nga tannafa?

………………………………..
………………………………..
………………………………..
…………………………………
………………………………...

iii) Omulwadde yafiira wa?

a) Waka
b) Mu ddwairo (Wandiika elinya ly'eddwaliro wano ………………………………….)

iv) Omulwadde yali akyamila empeke za Fluconazole nga tannafa?

a) Nedda
b) Ye, naye nga ayosaamu
c) Ye, era buli lunaku

v) Omulwadde yali akyamira empeke za ARV?

a) a) Nedda
b) Ye, naye nga ayosaamu
c) Ye, era buli lunaku

vi) Amulabilira yaberangawo emirundi emeka mu wiiki?

a) Ennaku 5-7 mu wiiki
b) Ennaku 2-4 mu wiiki
c) Obutaweza nnaku 2 mu wiiki

vi) Amulabiira yali amulinako kakwate ki?
a) Mukyala/mwami
b) Maama/taata
c) Waluganda
d) Kyanjawulo kwe'byo (kiwandike wano)
……………………………………………………..

viii) Omulwadde baddamu ko ne bamuwa ekitanda wakati w'okuweebwa ekitanda nga ajjanjabwa mulalama n'okumalayo doozi?

a) Ye
b) Nedda

ix) Oba ansa waggulu eri "ye", emirundi emeka?

a) Gumu
b) Emirundi 2
c) Emirundi 3
d) Gisukka ku esatu

4) Omulwadde bwaba akyali mulamu,

i) akyamira empeke za Fluconazole?

a)Nedda

b) Ye, naye nga ayosaamu

c) Ye, era buli lunaku

ii) Ansa waggulu bwebera “nedda”, lwaki omulwadde yali tamira fluconazole?

a) Omusawo yaliyimiriza
b) Eddagala teliliyo mu ddwaliro
c) Omulwadde yeyesalilawo

iii) Omulwadde akyamila eddagala ly’akawuka (ARV)?

a) Nedda
b) Ye, naye ayosaamu
c) Ye, era buli lunaku

iv) Omulwadde alina amulabilira?

a) Ye
b) Nedda

Ansa bweba “nedda” genda ku namba “vii”

v) alabilira omulwadde amulinako kakwaate ki?

a) Mukyala/mwami
b) Mama/taata
c) Waluganda
d) Kyanjawulo kwe’byo (kiwandike wano)
…………………………………………………..

vi) Amulabiira aberawo emirundi emeka mu wiiki?

a)Ennaku 5-7 mu wiiki

b)Ennaku 2-4 days a week

c)Ennaku teziweera 2 mu wiiki

vii) Omulwadde baddamu ko ne bamuwa ekitanda wakati w'okuwebwa ekitanda nga ajjanjabwa mulalama n'okumalayo doozi?

a) Ye
b) Nedda

viii) Oba "ye", emirundi emeka?

……………………………………

ix) Omulwadde yali azzemu okukola emirimu okuva lweyasibulwa mu ddwaliro?

a) Ye
b) Nedda

x) Ansa waggulu bwebera "ye", mirimu ki gy'akola?

…………………………………………………………………………………………
……………………....
