# Supplementary material for: Factors affecting mortality among HIV positive patients two years after completing recommended therapy for Cryptococcal meningitis in Uganda
Source: PLoS One. 2019 Jan 30;14(1):e0210287. doi: 10.1371/journal.pone.0210287 (PMC6353088; doi:10.1371/journal.pone.0210287)
Supplement: S1 Data extraction checklist — (PDF) [file pone.0210287.s004.pdf]

EVALUATION OF SURVIVAL OF HIV POSITIVE PATIENTS FOLLOWING STANDARD TREATMENT FOR CRYPTOCOCCAL MENINGITIS (CCM) IN UGANDA.

Data Extraction checklist from CryptoDex study

1) Patient ID
2) Date of birth
3) Number of years in school
4) Marital status
5) Date of HIV diagnosis
6) Last lumber puncture culture
7) Investigational product received (randomisation arm)
8) Date of starting ARVs
9) Date made week 10 in CryptoDex study
10) Quality of life at week 10
11) Date last known to be alive if lost to follow-up
